# A bioengineered immunocompetent human leukemia chip for preclinical screening of CAR T cell immunotherapy

**DOI:** 10.21203/rs.3.rs-2762929/v1

**Published:** 2023-04-21

**Authors:** Chao Ma, Huishu Wang, Lunan Liu, Jie Tong, Matthew T. Witkowski, Iannis Aifantis, Saba Ghassemi, Weiqiang Chen

**Affiliations:** 1 Department of Mechanical and Aerospace Engineering, New York University, Brooklyn, NY 11201, USA.; 2 Department of Biomedical Engineering, New York University, Brooklyn, NY 11201, USA.; 3 Perlmutter Cancer Center, NYU Grossman School of Medicine, New York, NY 10016, USA.; 4 Department of Pathology, NYU Grossman School of Medicine, New York, NY 10016, USA.; 5 Department of Pediatrics, University of Colorado Anschutz Medical Campus, Aurora, CO 80045, USA; 6 Center for Cellular Immunotherapies, Perelman School of Medicine at the University of Pennsylvania, Philadelphia, PA 19104, USA.; 7 Department of Pathology and Laboratory Medicine, Perelman School of Medicine at the University of Pennsylvania, Philadelphia, PA 19104, USA.

**Keywords:** Organ-on-a-Chip, Leukemia, CAR T cell therapy, Bone marrow niche, Immunity

## Abstract

Chimeric antigen receptor (CAR) T cell immunotherapy is promising for treatment of blood cancers; however, clinical benefits remain unpredictable, necessitating development of optimal CAR T cell products. Unfortunately, current preclinical evaluation platforms are inadequate due to their limited physiological relevance to humans. We herein engineered an organotypic immunocompetent chip that recapitulates microarchitectural and pathophysiological characteristics of human leukemia bone marrow stromal and immune niches for CAR T cell therapy modeling. This leukemia chip empowered real-time spatiotemporal monitoring of CAR T cell functionality, including T cell extravasation, recognition of leukemia, immune activation, cytotoxicity, and killing. We next on-chip modelled and mapped different responses post CAR T cell therapy, i.e., remission, resistance, and relapse as observed clinically and identify factors that potentially drive therapeutic failure. Finally, we developed a matrix-based analytical and integrative index to demarcate functional performance of CAR T cells with different CAR designs and generations produced from healthy donors and patients. Together, our chip introduces an enabling ‘(pre-)clinical-trial-on-chip’ tool for CAR T cell development, which may translate to personalized therapies and improved clinical decision-making.

## Introduction

Adoptive chimeric antigen receptor (CAR) T cell transfer has emerged as an encouraging immunotherapy for relapsed and refractory hematological malignancies, such as B cell acute lymphoblastic leukemia (B-ALL) [1–3], Diffuse large B cell lymphoma (DLBCL) [4,5], and multiple myeloma (MM) [6–8]. However, therapeutic outcomes vary across clinical trials, for example nearly half of patients yield to disease relapse with years of follow-up besides the side effects of cytokine release syndrome (CRS) and immune effector cell-associated neurotoxicity syndrome (ICANS) [9–11]. Several mechanisms including T cell dysfunction [12–14], surface antigen loss, down-regulation, and mutation [15–17], lineage swift [18,19], and impaired death ligand [20] that may lead to treatment failures have been reported, yet there are currently no effective molecular biomarkers that can predict patient responses. To prevent these setbacks and risks during CAR T cell development and clinical treatment, biopharmaceutical researchers need a preclinical testing platform that delivers human relevant results.

Current strategies for preclinical assessment of CAR T cell function rely on *in vitro* and *in vivo* models [21–23]. Conventional *in vitro* assays such as two-dimensional (2D) cell co-cultures and 3D tumor spheroids/organoids demonstrate limited predictive value due to their lack of tumor associated stroma and/or immune components, thus are just useful for CAR T cell development at early-stage. *In vivo* animal models have been widely established for preclinical cancer research, however, most of them are either immunodeficient or differing from host immunity, dictating the pursuit of humanized models [21–24]. Critically, current humanized animal models are painstaking due to extensive preparation and subsequent month-long experiments and thwart real-time and in situ monitoring of CAR T cell response [9]. It thus demands a reliable precision immuno-oncology tool that enables rapid, in-depth evaluation of CAR T cell therapy within a human pathophysiologically relevant context [25–27].

In this study, we developed an *ex vivo* organotypic and immunocompetent human leukemia microphysiological system exemplifying a *bond fide* leukemia bone marrow niche integrative of both stroma and immune cells. Through on-chip real-time live cell imaging, comprehensive proteomic and secretomic profiling, and high-throughput single cell mRNA sequencing (scRNA-seq), we precisely captured systematical and spatiotemporal dynamics of CAR T cell treatment, ranging from T cell extravasation, immune activation, and T cell cytotoxicity to T cell killing. Also, our bioengineered chip accurately validated functional performance of CAR T cell products with different designs and generations of CAR and those produced from cancer patients. Lastly, we developed a matrix-based analytical and functional index to comprehensively delineate optimal CAR T cell. This unique preclinical platform enables a precise, reliable, and systematic evaluation of CAR T cell therapy, which can be readily extended to evaluate many other immunotherapies for different blood cancers as well as solid tumors and beyond.

## Results

### Engineering an *ex vivo* immunocompetent human leukemia chip

**Extended Data Fig. 1.**
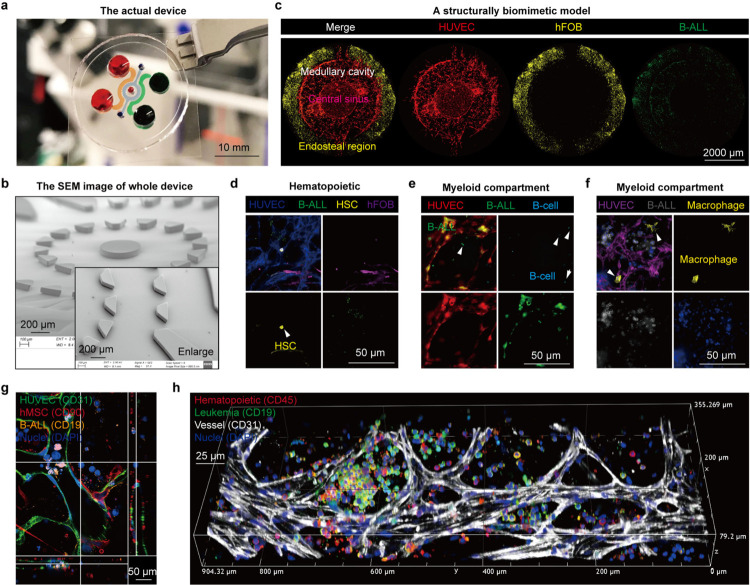
Design and fabrication of bone marrow niche chip. (**a**) The actual chip of human leukemia bone marrow where chambers and channels were filled with food dyes. Scale bar, 10 mm. (**b**) The scanning electron microscopy images of detailed design of the microfluidic chip. Scale bar, 200 μm. (**c**) The whole scanning of the bone marrow niche on-chip, where RFP-expressing HUVCEs form a vascular network (red), hFOB1.19 osteoblast (yellow) and fibroblast establish an endosteal region, and Reh B-ALL cells (green) sparsely distributing throughout the niche. Scale bar, 2000 μm. (**d**) Representative images showing the presence of hematopoietic stem cells (CD34^+^) in yellow in the endosteal niche. Scale bar, 50 μm. (**e**) Representative images showing the presence of B cell (CD19^+^ without GFP) and B-ALL blasts (CD19^+^ and GFP). Scale bar, 50 μm. (**f**) Representative images showing the presence of macrophages (CD68^+^) in yellow. Scale bar, 50 μm. (**g**) The 3D cross-section view of the stromal compartment. vascular cells in green (CD31^+^), bone marrow stem/stromal cells in red (CD90^+^), leukemia blasts in yellow (CD19^+^), and nuclei in blue (DAPI). Scale bar, 50 μm. (**h**) The 3D view of the vascular niche. Hematopoietic cells in red (CD45^+^), leukemia blasts in green (CD19^+^), vessel in white (CD31^+^), and nuclei in blue (DAPI). Scale bar, 25 μm. Representative images were from one of at least three technical replicates (n≥3).

**Extended Data Fig. 2.**
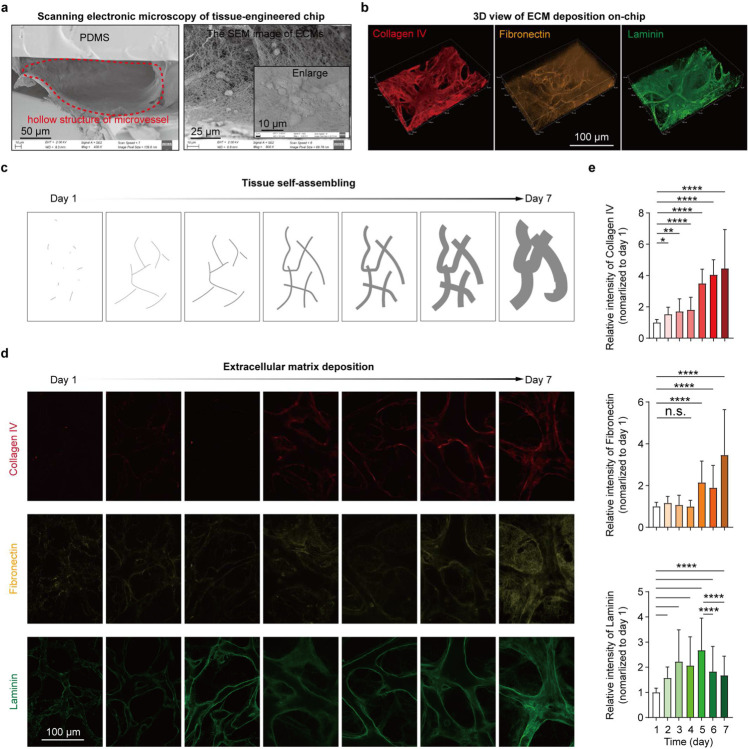
Cell self-assembling and tissue remodeling. (**a**) Scanning electronic microscopy images of tissue-engineered chip after 7-day culture. The hollow structure of microvessel formed during on-chip culture (red dash line, left) and extracellular matrix where bone marrow cells are embedded (right). Scale bars, 50 μm (left), 25 μm (right), and 10 μm (inset). (**b**) Representative images showing 3D view of key ECM components (collagen IV, fibronectin, and laminin) deposited by bone marrow niche cells on-chip after 7-day culture. Scale bar, 100 μm. (**c**) The schematic showing cell self-assembling. (**d**) Representative images showing time-lapse ECM deposition by bone marrow cells during 7-day culture. Scale bar, 100 μm. (**e**) Quantification of ECM deposition. Collagen IV (top), Fibronectin (middle), and Laminin (bottom). Images were collected from at least five technical replicates (n≥5) and processed with CellProfiler (version 4.1.3). Nonparametric Krusakl-Wallis’s test. Data with statistical significance are as indicated: *P < 0.05, **P < 0.01, ***P < 0.001, ****P < 0.001, and n.s., not significant (P > 0.05).

**Extended Data Fig. 3.**
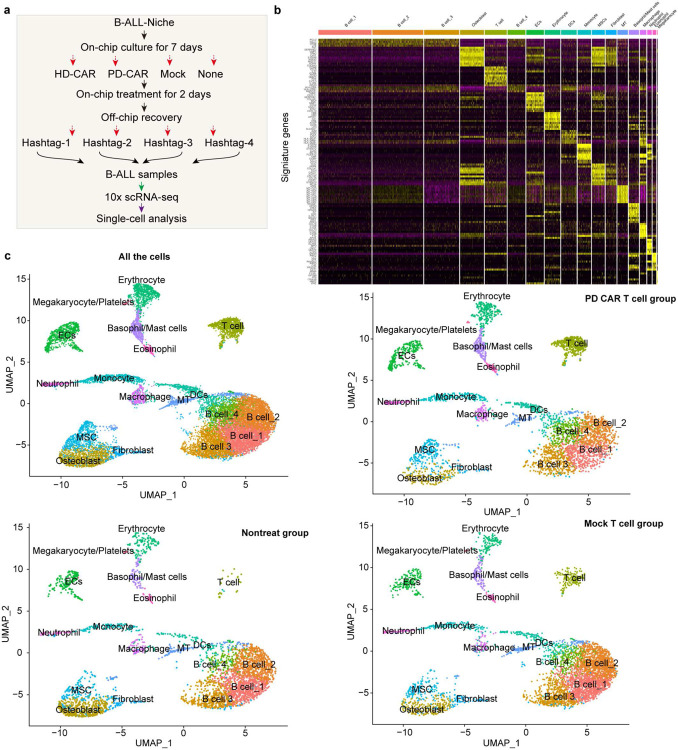
scRNA-seq mapping of engineered bone marrow niche. (**a**) The schematic showing the sample preparation for scRNA-seq. (**b**) Signature genes (top 20) of each cluster identified. (**c**) The UMAP presentation of different cell populations from different samples, where bone marrow niche chips were treated with HD CAR T cell, PD CAR T cell, Mock T cell, or left non-treated, corresponding to Fig. 1 and Fig. 2.

**Fig. 1.**
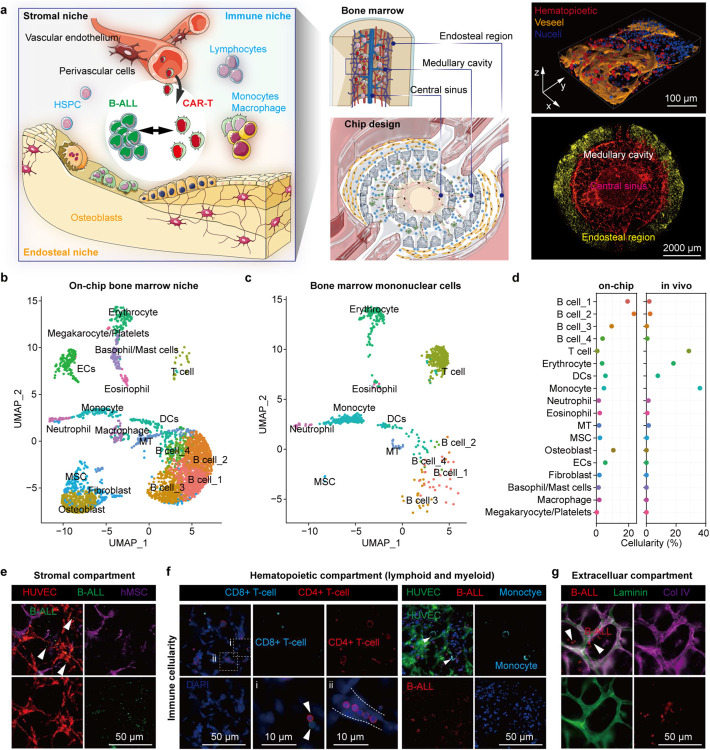
Bioengineering an *ex vivo* organotypic and immunocompetent bone marrow niche chip. (**a**) The compartmentalized bone marrow chip (middle) was populated with human bone marrow cells (right) to replicate the *in vivo* counterpart (left). The whole scan of the leukemia bone marrow chip (bottom right) with central sinus (RFP-HUVECs in red), medullary cavity, and endosteum (DiD labelled osteoblasts in yellow). The 3D view of perivascular niche (top right) in bone marrow with hematopoietic cells (CD45^+^ in red), vascular cells (CD31^+^ in yellow), and nuclei in blue (DAPI). Scale bars, 50 μm (top right) and 2000 μm (bottom right). (**b**) scRNA-seq profiling of the bone marrow cellularity on-chip, highlighting the presence of most hematopoietic (immune) and non-hematopoietic (bone marrow stroma) cells. (**c**) scRNA-seq profiling of primary bone marrow mononuclear cells, which was comparatively mapped to that of on-chip bone marrow niche. (**d**) The cellularity. In addition to the bone marrow stromal cell populations seeded to build stromal environment, macrophage, basophil/mast cells, and megakaryocyte/platelets, were generated on-chip during culture. (**e**) The presence of stromal compartment. HUVECs in red (RFP), Reh B-ALL cells in green (GFP), mesenchymal cells in purple (DiD labeling). Scale bar, 50 μm. (**f**) The presence of hematopoietic cells. Lymphoid (left): CD8^+^ T cells in cyan and CD4^+^ T cells in red. Scale bar, 50 μm. Scale bars for insets (i) and (ii), 10 μm. Myeloid (right): monocyte (CD14^+^) in cyan, HUVECs (CD31^+^) in green, and Reh B-ALL cells (CD19^+^) in red. Scale bar, 50 μm. (**g**) The deposition of ECMs such as laminin (green) and collagen IV (purple). Reh B-ALL cells in red. Scale bar, 50 μm. (**e**,**g**) Representative images were from one of at least three technical replicates (n≥3).

To fill the biological and technical gaps in delivering an in-depth evaluation of CAR T cell therapy dynamics within a human relevant context, we established an *ex vivo* vascularized and immunocompetent human leukemia bone marrow niche on-chip. Anatomically, leukemia bone marrow is a multi-compartmental milieu divided into regions of central sinus, medullary cavity, and endosteum, where dysfunctionally orchestrated interactions between leukemia blasts and hematopoietic and non-hematopoietic niche cells in 3D extracellular matrices (ECMs) maintain disease progression and promote therapy resistance (Fig. 1a, **left**). To replicate the *in vivo* tissue architecture *in vitro*, our microfluidic chip is designed with three interconnected structural regions, including a central sinus linked to concentric medullary cavity encircled by outer endosteal region (Fig. 1a, **middle** and Extended Data Fig. 1a,b) and fabricated using replica molding [28–30]. The chip was populated with human hematopoietic cells (bone marrow mononuclear cells), leukemia blasts, stromal cells (vascular cells, mesenchymal stem cells, osteoblasts, and fibroblasts) within compartmentalized fibrin hydrogels inside which the seeded bone marrow stromal cells rebuilt and supported the three concentric tissue structures (Methods). We next exploited the *de novo* vascularization process of vascular cells under guidance from intrinsic and extrinsic cues such as vascular growth factors and ECMs to vascularize the tissue structure [31–33]. In brief, the cell-laden chip was cultured with a myriad of cytokines (maintaining hematopoietic cells and promoting vasculogenesis) over 7 days during which vascular cells self-assembled into perfusable vascular network aligned by stromal cells throughout the 3D matrix (Fig. 1a, **right** and Extended Data Fig. 1c–h). Hematopoietic cells and leukemia blasts sparsely distributed within the peri-/vascular and endosteal niches, which well matched the *in vivo* cellular localization and composition (Extended Data Fig. 1d–h). Notably, bone marrow stromal cells (mostly vascular cells) progressively deposited supplementary ECMs such as laminin, fibronectin, and collagen IV around the vasculature and throughout the niche (Extended Data Fig. 2), highlighting cell-autonomous remodeling of engineered tissue into a more biomimetic one [34–36]. To validate the biomimicry of bioengineered bone marrow niche to its *in vivo* counterpart, we next utilized scRNA-seq technique to comparatively map cellularity in the sample of cells harvested from our chip and matched sample of unloaded fresh bone marrow mononuclear cells (Fig. 1b,c Extended Data Fig. 3, and Methods). The scRNA-seq results validated that our bone marrow chip, even after over 7-day culture, maintained an enriched cellularity with hematopoietic and stromal cells, comparable to that of the *in vivo* bone marrow (Fig. 1d), keeping with recent scRNA-seq studies [28,37–40]. We further corroborated this observation with immunostaining and found most types of lymphoid (e.g., CD8^+^ and CD4^+^ T cells) and myeloid (e.g., CD14^+^ monocytes and CD68^+^ macrophages) cells were well maintained on-chip along with the presence of vascular network (Fig. 1e–g and Extended Data Fig. 1). Together, our tissue-engineered leukemia chip recreates *in vitro* microarchitectural organization and pathophysiological signature of both human leukemia bone marrow stroma and immune niches *in vivo*.

### Modeling CAR T cell therapy on-chip

**Extended Data Fig. 4.**
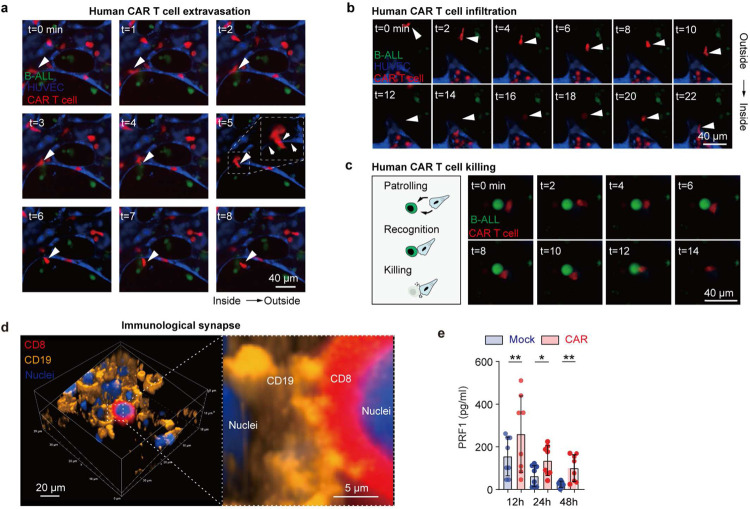
Monitoring CAR T cell dynamics on-chip. (**a**) Representative images showing CAR T cell extravasation. T cell in red, Reh B-ALL in green, and vessel (HUVECs) in bule. Scale bar, 40 μm. (**b**) Representative images showing CAR T cell infiltration. T cell in red, Reh B-ALL in green, and vessel (HUVECs) in bule. Scale bar, 40 μm. (**c**) Representative images showing the process of killing a B-ALL blast (green) by a CAR T cell (red). Scale bar, 40 μm. (**d**) A representative 3D image showing the intercellular contact between CAR T cell (CD8 in red) and leukemia blasts (CD19 in yellow) where intercellular synapse is formed and cytolytic granules are released. Scale bars, 40 μm and 5 μm (inset). (**e**) Secretion of perforin (PRF1) on-chip from leukemia chips treated with CAR T cell (CAR) and Mock T cell (Mock) at different time points (12 hours, 24 hours, and 48 hours). Data was collected from four independent experiments (n=4). Unpaired Student’s t-test, mean ± SD. Data with statistical significance are as indicated: *P < 0.05, **P < 0.01, ***P < 0.001, and ****P < 0.0001.

**Extended Data Fig. 5.**
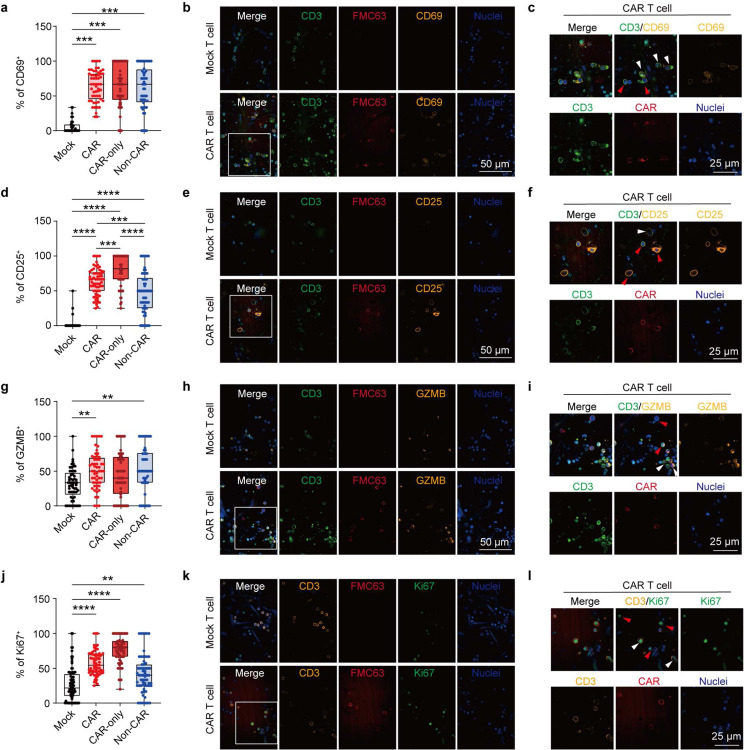
Activation of bystander T cells during CAR T cell activation on-chip. (**a-c**) Quantification of T cell activation with surface expression of (**a-c**) CD69 and (**d-f**) CD25 and intracellular expression of (**g-i**) GZMB and (**j-l**) Ki67 in all CD3^+^ T cell from CAR T cell treated group (**CAR**) and Mock T cell treated group (**Mock**). CAR-expressing T cell (**CAR-only**, CD3^+^FMC63^+^) and non-CAR expressing T cell (**non-CAR**, CD3^+^FMC63^−^). Scale bars, 100 μm and 25 μm. Data was collected from four technical replicates (n=4). One-way analysis of variance (ANOVA) followed by Tukey’s post hoc test. Data with statistical significance are as indicated: *P < 0.05, **P < 0.01, ***P < 0.001, ****P < 0.0001, and n.s., not significant (P > 0.05).

**Fig. 2.**
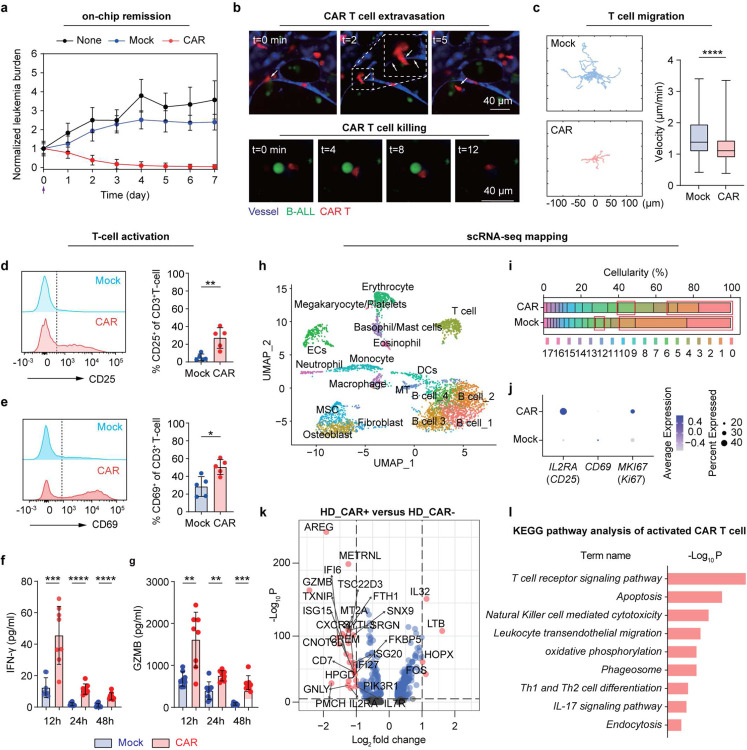
Monitoring CAR T cell dynamics in the leukemia bone marrow niche on-chip. (**a**) Leukemia burdens of leukemia chips treated either with CAR T cell (CAR, red line), Mock T cell (Mock, blue line) or left untreated (None, dark line). Quantitative data was collected from four technical replicates (n≥4). (**b**) Representative images of at least three technical replicates (n≥3) showing CAR T cell extravasation (top) and killing of B-ALL blast (bottom). CAR T-cell in red, leukemia blast in green, vessel in blue. Scale bar, 40 μm. (**c**) Comparison of T cell migration dynamics. Representative trajectories of 10 Mock T cells (top left) and 10 CAR T cells (bottom left) monitored for a 4-hour period after on-chip infusion for 2 days. Quantitative data (right) was collected from four technical replicates (n≥4) with >100 T cells. Unpaired Student’s t-test, mean ± SD. Data with statistical significance are as indicated: ****P < 0.0001. (**d**,**e**) Flow cytometry measurement of surface expressions of T cell activation markers CD25 (**d**) and CD69 (**e**) on CAR T cell and mock T cell. Data was collected from five independent experiments (n=5). Unpaired Student’s t-test, mean ± SD. Data with statistical significance are as indicated: *P < 0.05 and **P < 0.01. (**f**,**g**) ELISA measured secretions of INF-γ (**f**) and GZMB (**g**) from leukemia chips respectively treated with CAR T cell (CAR) and Mock T cell (Mock) at different time points (12 hours, 24 hours, and 48 hours). Data was collected from four independent experiments (n=4). Unpaired Student’s t-test, mean ± SD. Data with statistical significance are as indicated: *P < 0.05, **P < 0.01, ***P < 0.001, and ****P < 0.0001. (**h**) scRNA-seq mapping of bone marrow niche treated with CAR T cell on-chip for 2 days. (**i**) Cellularity of samples harvested from bone marrow chip treated with CAR T cell or Mock T cell. Red boxes indicate CD19 expressing populations. (**j**) Dot plot representation of mRNA expression levels of *IL2RA* (*CD25*), *CD69*, and *MKI67* (*Ki67*) in CAR T cell and Mock T cell. (**k**) Analysis of Differentially Expressed Genes (DEG) in CAR T cells before and after activation (on-chip). (**l**) KEGG pathway analysis revealed enhanced signaling pathways in CAR T cells before and after activation (on-chip).

We then modeled anti-CD19 CAR T cell therapy on-chip within a human pathophysiologically relevant context and monitored CAR T cell dynamics in a real-time manner (Fig. 2, Extended Data Fig. 4, Extended Data Fig. 5, and Methods). We first infused 10,000 (effector:tumor = 1:1) of second generation (2nd-gen) anti-CD19 4-1BBζ-CAR T cells (hereafter CAR T cell, unless stated otherwise) per chip into the perfusable vessels from central sinus and quantified the count of pre-seeded leukemia blasts on-chip (GFP-expressing Reh, unless stated otherwise) daily for over 7 days. The results showed CAR T cell killed ~70% of leukemia blasts on-chip after 3 days and achieved complete eradication (>99%) of leukemia blasts around 7 days, reminiscent of clinical remission, whereas leukemia chips either treated with non-transduced T cell (referred as to Mock T cell) or left untreated (referred as to None) showed no control over leukemia progression (Fig. 2a). Then, we longitudinally charted the migratory behaviors of CAR T cell (labeled with DiD dye) in the medullary cavity region (vessel formed by RFP-expressing HUVECs) for 12 hours with fluorescent live imaging after infusion for 2–4 days (Fig. 2b and Extended Data Fig. 4a–c). We found that CAR T cells actively patrolled the leukemia bone marrow niche such as extravasated out vessel (Fig. 2b and Extended Data Fig. 4a) and infiltrated into the perivascular area (Extended Data Fig. 4b). Notably, we captured in real-time the whole process of CAR T cell killing of leukemia at single-cell level where a single CAR T cell moved ameboidly to reach, recognize, and kill an individual leukemia blast (Fig. 2b and Extended Data Fig. 4c). The migration trajectory from a 4-hour observation further demonstrated different patterns between CAR T cell (mCherry expressing) and Mock T cell (DiD labeling), where CAR T cell actively patrolled and intermittently arrested at leukemia blasts with a mean velocity of 1.213 μm/min, while Mock T cell continuously patrolled at a mean velocity of 1.545 μm/min (Fig. 2c). This reduced velocity of CAR T cell may be due largely to its recognition of CD19^+^ leukemia blasts and contact with the latter to form an intercellular synapse (Extended Data Fig. 4d), where cytolytic granules for example are released.

### Examining CAR T cell functionality on-chip

**Extended Data Fig. 6.**
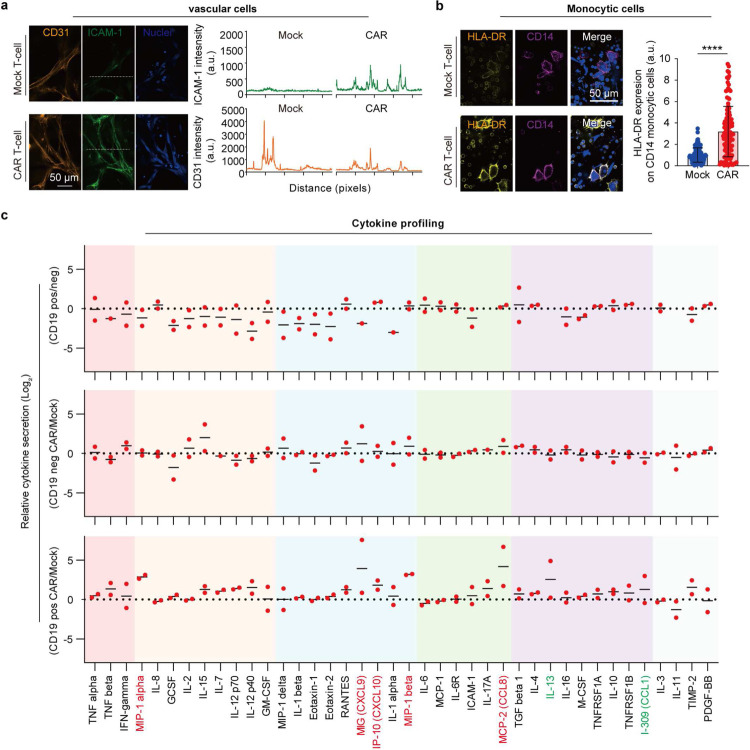
CAR T cell activation triggers systematic immune response from leukemia bone marrow niche. (**a**) Representative images from one of three technical replicates (n=3) showing ICAM-1 (green) expression on vascular cells (CD31^+^ in yellow) from leukemia chips respectively treated Mock T cell (top left) and CAR T cell (top bottom) for 2 days. Quantification data of ICAM-1 and CD31 intensity, corresponding to the white dash lines. (**b**) Representative images showing HLA-DR expression on monocytic cells (CD14^+^) from leukemia chips respectively treated Mock T cell and CAR T cell for 2 days. Scale bar, 50 μm. Data was collected from four independent experiments (n=4). Unpaired Student’s t-test, mean ± SD. Data with statistical significance are as indicated: ****P < 0.0001. (**c**) Profiling of cytokine secretion from leukemia chips that were established with CD19^+^ (pos) and CD19^−^ (neg) leukemia blast and treated with CAR T cell (CAR), Mock T cell (Mock) or left untreated (None). Data was collected from two independent experiments.

We next characterized CAR T cell functionality, such as T cell activation, cytotoxicity, and proliferation, after its interaction with leukemia blasts in niche on-chip for 2 days. Compared to Mock T cell, CAR T cell significantly enhanced surface expression of T cell activation markers, CD25 and CD69 (Fig. 2d,e and Extended Data Fig. 5a–f), secretion of cytotoxicity-related cytokines, interferon-γ (IFN-γ, Fig. 2f), granzyme B (GZMB, Fig. 2g and Extended Data Fig. 5g–i), and perforin (PRF1, Extended Data Fig. 4e), and intracellular expression of proliferation marker Ki67 (Extended Data Fig. 5j–l). Interestingly, we noted that non-CAR expressing T cell from CAR T cell treated chips also showed enhanced expression of T cell activation, cytotoxicity, and proliferation related markers (Extended Data Fig. 5), validating previous *in vivo* observation of bystander effects during CAR T cell therapy [41]. We hypothesized that CAR T cell treatment can induce systematic immune response from the leukemia bone marrow niche on-chip. Upon infusion of CAR T cell, vascular cells enhanced surface expression of intercellular adhesion molecule-1 (ICAM-1), a molecule supporting T cell extravasation (Extended Data Fig. 6a), while CD14^+^ monocytic cells increased surface expression of HLA-DR, an MHC-II molecule for antigen presentation (Extended Data Fig. 6b), highlighting CAR T cell induced systematic inflammation in niche. Meanwhile, we observed an excessive on-chip production of cytokines, such as immune stimulatory cytokines (e.g., GM-CSF), chemokines (e.g., RANTES), inflammatory cytokines (e.g., MCP-2), and regulatory cytokines (e.g., Interleukin(IL)-10), among many others (Extended Data Fig. 6c). This augmentation of cytokine secretion presented only in chips where CAR T cell interacted with CD19^+^ leukemia blasts but not in those where Mock T cell interacted with CD19^+^ leukemia blasts or CAR T cell with CD19 knockout (CD19^−^) ones (Extended Data Fig. 6c), confirming the specificity and efficacy of anti-CD19 CAR T cell therapy.

**Extended Data Fig. 7.**
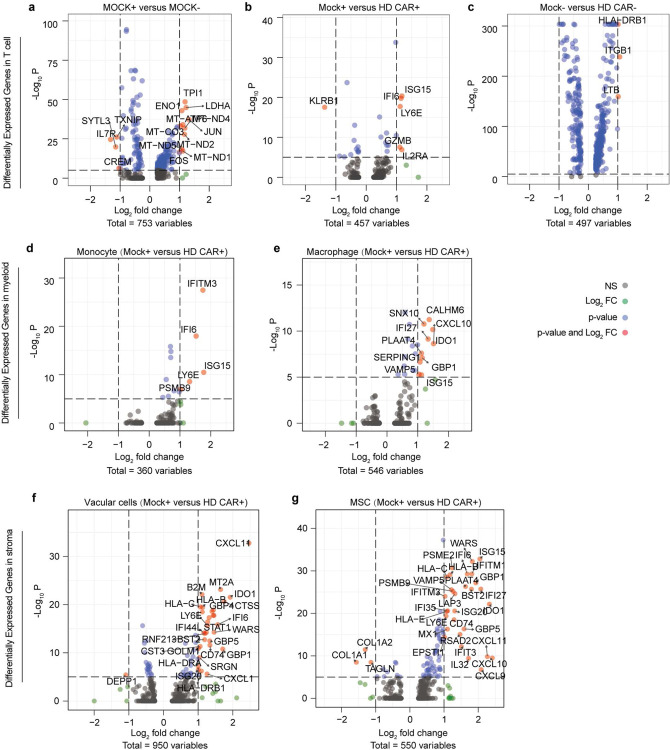
scRNA-seq mapping of leukemia chips treated with healthy donor (HD) derived CAR T cell. (**a**) Analysis of Differentially Expressed Genes (DEG) in Mock T cell before (Mock−) and after (Mock+) interaction with leukemia blasts on-chip for 2 days. (**b**,**c**) DEG analysis between CAR T cell (HD CAR) and Mock T cell after (**b**) and before (**c**) interaction with leukemia blasts on-chip for 2 days. (**d-g**) DEG analysis of monocyte (**d**), macrophage (**e**), vascular cells (**f**), and hMSC stromal cells (**g**) from leukemia chips treated with CAR T cell (HD CAR+) or Mock T cell (Mock+) for 2 days.

**Extended Data Fig. 8.**
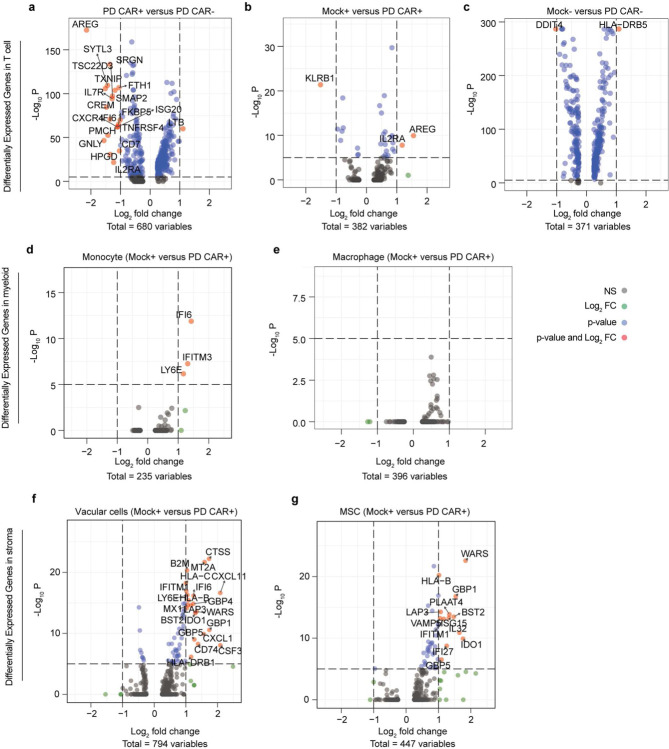
scRNA-seq mapping of leukemia chip treated with patient derived (PD) CAR T cell. (**a**) Analysis of Differentially Expressed Genes (DEG) in Mock T cell before (Mock−) and after (Mock+) interaction with leukemia blasts on-chip for 2 days. (**b**,**c**) DEG analysis between CAR T cell (PD) and Mock T cell after (**b**) and before (**c**) interaction with leukemia blasts on-chip for 2 days. (**d-g**) DEG analysis of monocyte (**d**), macrophage (**e**), vascular cells (**f**), and hMSC stromal cells (**g**) from leukemia chips treated with CAR T cell (PD CAR+) or Mock T cell (Mock+) for 2 days.

Following this, we applied scRNA-seq technique to comparatively dissect the leukemia bone marrow niches treated with CAR T cell or Mock T cell on-chip for 2 days (Fig. 2h, Extended Data Fig. 3, and Extended Data Fig. 7). First, scRNA-seq results reaffirmed reduced population of CD19 expressing clusters in niche upon treatment with CAR T cell but not Mock T cell (Fig. 2i). We found that CAR T cell, compared to Mock T cell, enhanced mRNA expression of T cell activation and proliferation related genes such as *IL2RA*, *CD69*, and *MKI67* (Fig. 2j and Extended Data Fig. 7a–c), validating aforementioned observations with on-chip immunostaining. Moreover, activated CAR T cell increased mRNA expression of cytotoxicity related genes such as *GZMB* and *GNLY* (Fig. 2k) and immune response signaling pathways such as T cell receptor signaling pathway, Th1 and Th2 cell differentiation, Natural Killer cell mediated cytotoxicity, and leukocyte transendothelial migration (Fig. 2l). Remarkably, CAR T cell triggered systematic responses from both immune (Extended Data Fig. 7d,e) and stromal cells (Extended Data Fig. 7f,g) with altered transcriptional cascades, such as enhanced mRNA expression of interferon-induced transmembrane protein (IFITM) family members. We then expanded the scRNA-seq analysis to a leukemia patient-derived CAR T cell (PD CAR) that presented a similar profile of functional dynamics upon activation on-chip and such activated PD CAR T cell also induced a systematic response across most cell types in the leukemia bone marrow niche (Extended Data Fig. 8). These results demonstrated the capacity of our leukemia chip for preclinical evaluation of functional dynamics of CAR T cell and its interaction with the leukemia bone marrow stromal and immune cells.

### Mapping heterogeneous CAR T cell response scenarios

**Extended Data Fig. 9.**
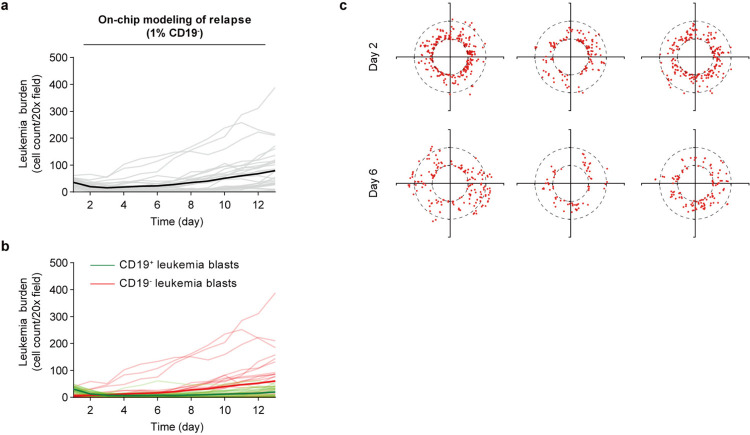
Modeling leukemia relapse post CAR T cell therapy. (**a**) Leukemia burden under CAR T cell therapy in the leukemia bone marrow niche spiked with 1% CD19^−^ leukemia blasts. Data was collected from three independent experiments each with 2–3 chips, where each chip has four random fields quantified (n=32 images). (**b**) The counts of CD19^+^ (green lines) and CD19^−^ (red lines) leukemia blasts in the leukemia bone marrow niche spiked with 1% CD19^−^ leukemia blasts, corresponding to (**a**). (**c**) Dynamic distribution of CAR T cells in remission, resistance, and relapse scenarios on day 2 and 6, corresponding to Fig. 3c. Each dot represents a CAR T cell. Black dash circles indicated the three concentric regions, central sinus, medullary cavity, and endosteum. Representative data was from one of three technical replicates (n=3).

**Fig. 3.**
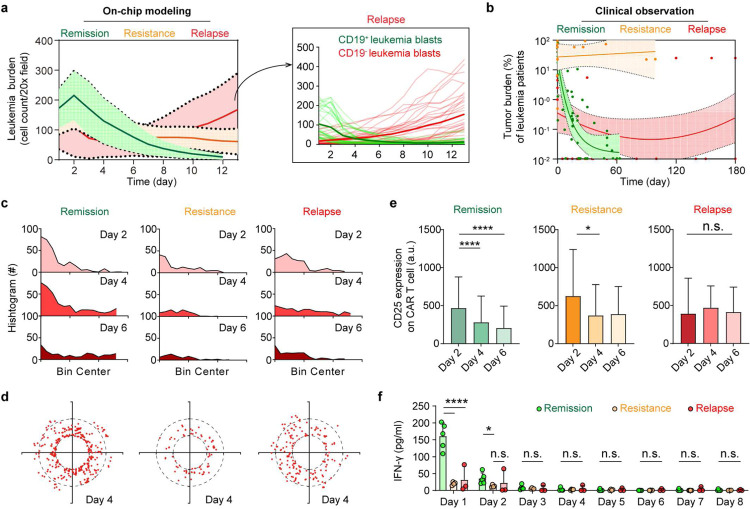
Modeling different leukemia response scenarios post CAR T cell therapy. (**a**) On-chip monitoring of tumor burdens in leukemia chips treated with CAR T cells under response scenarios (i.e., remission, resistance, and relapse). Data was collected from three independent experiments each with 2–3 chips, where each chip has four random fields quantified (n=32 images). Inset demonstrated the counts of CD19^+^ (green lines) and CD19^−^ (red lines) leukemia blasts in the leukemia bone marrow niche spiked with 5% CD19^−^ leukemia blasts (relapse scenario). (**b**) Clinical data showing tumor burdens of leukemia patients during CAR T cell therapy. Data was pooled and adopted from Liu et al [48], where different clinical studies with 209 patients were collected and unified. Non-linear fittings were applied to remission and relapse scenarios and linear regression was applied to resistance response with 95% confidence intervals. (**c**) Histogram showing the distance of CAR T cell to the center of the central sinus on-chip in remission, resistance, and relapse scenarios at day 2, 4, and 6. Representative data was from one of three technical replicates. (**d**) Dynamic distribution of CAR T cell in remission, resistance, and relapse scenarios at day 4, corresponding to (**c**). Each dot represents a CAR T cell. Black dash circles indicated the three concentric regions, central sinus, medullary cavity, and endosteum. Representative data was from one of three technical replicates (n=3). (**e**) Surface expression of CD25 on CAR T cell under different response scenarios. Data was collected from three independent experiments (n=3). Unpaired t test, mean ± SD. Data with statistical significance are as indicated: *P < 0.05, **P < 0.01, ***P < 0.001, ****P < 0.0001, and n.s., not significant (P > 0.05). (**f**) ELISA measured secretion of IFN-γ from leukemia chips of different response scenarios under CAR T cell therapy at different time points. Data was collected from at least three independent experiments (n≥3). Data with statistical significance are as indicated: *P < 0.05, **P < 0.01, ***P < 0.001, ****P < 0.0001, and n.s., not significant (P > 0.05).

Our understanding of the dynamic changes in the leukemia bone marrow microenvironment during CAR T cell therapy responses such as resistance or effective killing are unclear, which may prevent unleashing its full therapeutic potential [42,43]. We herein modelled the processes of leukemia resistance and relapse after CAR T cell therapy using our bioengineered leukemia chip and mapped the underlying molecular and cellular changes (Fig. 3). Clinical diagnosis by cytomorphology sets leukemia burden in bone marrow below 5% as remission while above 25% as relapse or resistance after treatment, though other assays such as PCR and flow cytometry take a lower percentage (0.01%−0.1%) as remission [44–47]. We here followed the standard set by cytomorphology as it could allow us to quantify the number of leukemia blasts in a single chip continuously during CAR T cell therapy with time-lapse imaging. To on-chip replicate relapse scenario from pre-existing minor CD19^−^ clones, we spiked CD19^−^ leukemia blasts (5% or 1%) into the total leukemia population when preparing leukemia chips, while to mimic resistance (i.e., refractory) scenario, we infused 2,500 CAR T cells (effector:tumor = 1:4) per chip compared to 10,000 in the remission and relapse scenarios (Methods). Then, we monitored leukemia burden chronologically by fluorescence imaging of on-chip CD19^+^ leukemia blasts with GFP signal and CD19^−^ ones with mCherry signal every day for over 14 days. The on-chip response curves showed that 2,500 CAR T cells mostly failed to control leukemia progression (Fig. 3a), replicating clinical refractory cases possibly caused by insufficient expansion/persistence of patient CAR T cell products. Likewise, 10,000 CAR T cells eradicated most CD19^+^ leukemia blasts (initial response) but spared CD19^−^ ones which thus expanded unrestrainedly, reminiscent of clinical relapse cases driven by pre-existing CD19^−^ populations (surface antigen loss) and selective pressure from CAR T cell (Extended Data Fig. 9a,b). We next pooled and plotted clinical data of tumor burdens from 209 leukemia patients during CAR T cell therapy at different time points collected in our recent study [48]. As shown in Fig. 3b, some patients achieved remission with decreased leukemia burden (remission cases), where some showed continuous leukemia progression (resistance cases) and others achieved initial remission but unfortunately leukemia reemerged several months (relapse cases) post CAR T cell therapy, confirming that our on-chip modelled different response scenarios mirrored to some extent the dynamic patterns of clinical scenarios. We then tracked migration dynamics of CAR T cell in different response scenarios and found that CAR T cells from both remission and relapse conditions presented a pattern of wide distribution from medullar cavity to endosteum where those from resistance/refractory condition had limited range of distribution during CAR T cell therapy on-chip (Fig. 3c,d and Extended Data Fig. 9c). Intriguingly, CAR T cell demonstrated decreased surface expression of T cell activation marker CD25 in remission and resistance groups but not in relapse group (Fig. 3e). To identify molecular changes across different response scenarios, we further monitored dynamics of cytokine secretion (i.e., IFN-γ) and found that IFN-γ was highest in remission scenarios compared to that in resistance and relapse ones (Fig. 3f). These results together reproduced clinical resistance and relapse scenarios, offering us an experimental model to real-time dissect CAR T cell therapy within the whole disease spectrum.

### Validating functional performance of CAR T cell products

**Extended Data Fig. 10.**
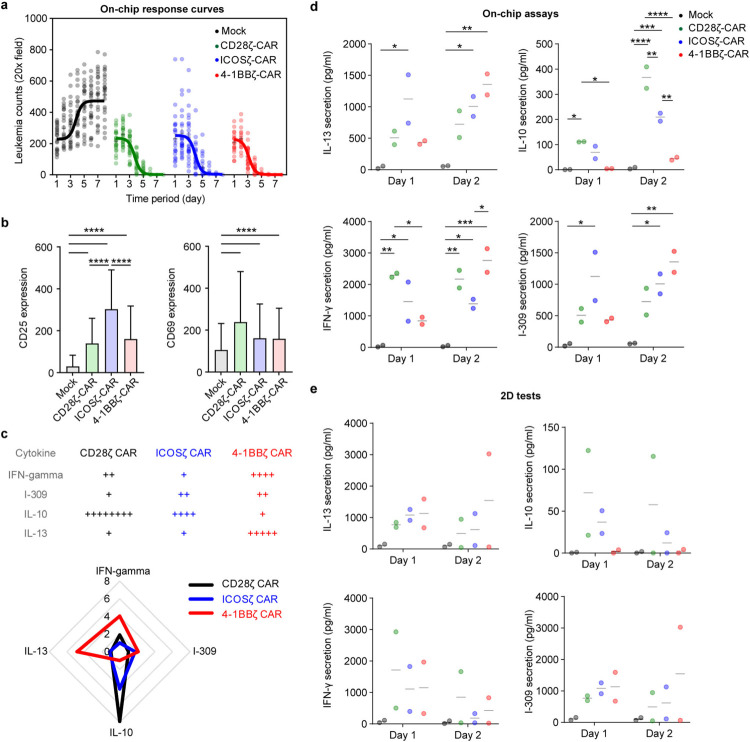
Comparative analysis of 2nd-gen CAR T cell products. (**a**) On-chip response curve under treatment of different 2nd-gen CAR T cells. Data was collected from two independent experiments, and each had 2 or 3 chips. (**b**) CD25 (left) and CD69 (right) expression on different 2nd-gen CAR T cells activated by interaction with leukemia on-chip for 2 days. Data was collected from two independent experiments, and each had 2 or 3 chips. Kruskal-Wallis’ test. Data with statistical significance are as indicated: *P < 0.05, **P < 0.01, ***P < 0.001, ****P < 0.0001 and n.s., not significant (P > 0.05). (**c**) Radar mapping of cytokine secretion from leukemia chip treated with different 2nd-gen CAR T cells for 2 days. Data was normalized to that of Mock T cell, corresponding to Extended Data Fig. 11. (**d**,**e**) ELISA measurement of IL-13, IL-10, IFN-γ, and I-309 secretion from (**d**) on-chip and (**e**) 2D co-culture conditions where different 2nd-gen CAR T cells interacted with leukemia blasts for 2 days. Data was from CAR T cell and Mock T cell of two healthy donors (ND164 and ND365). Two-way analysis of variance (ANOVA) followed by Tukey’s post hoc test. Data with statistical significance are as indicated: *P < 0.05, **P < 0.01, ***P < 0.001, ****P < 0.0001 and n.s., not significant (P > 0.05).

We next leveraged our leukemia chip to preclinically assess the therapeutic potency of CAR T cell products generated with different designs/generations and from both healthy donors and cancer patients. We first validated our recently developed 2nd-gen anti-CD19 CAR T cells with different designs of CAR, i.e., CD28ζ-CAR, ICOSζ-CAR, and 4-1BBζ-CAR, and found that these CAR T cells all achieved remission on-chip at a dose of 10,000 CAR T cells (Extended Data Fig. 10a). Also, these CAR T cells enhanced surface expression of CD25 and CD69, though at different levels, upon activation in niche on-chip, compared to that of mock T cell (Extended Data Fig. 10b). Intriguingly, cytokine profiling from on-chip tests demonstrated that 4-1BBζ-CAR secreted more IL-13 (a Th2 cytokine), and CD28ζ-CAR and ICOSζ-CAR secreted more IL-10, whereas this is not the case for 2D tests with respective co-cultures of CAR T cell and leukemia blast (Extended Data Fig. 10c–e and **Fig. S11**). Such difference may be regulated by intrinsic and extrinsic cues in systematic immune response as previous scRNA-seq studies identified Th2 signaling deficiency as an indicator of antigen-positive leukemia relapse post CAR T cell therapy [49,50] and clinical observations showed that 4-1BBζ-CAR persisted longer than CD28ζ-CAR [51].

**Fig. 4.**
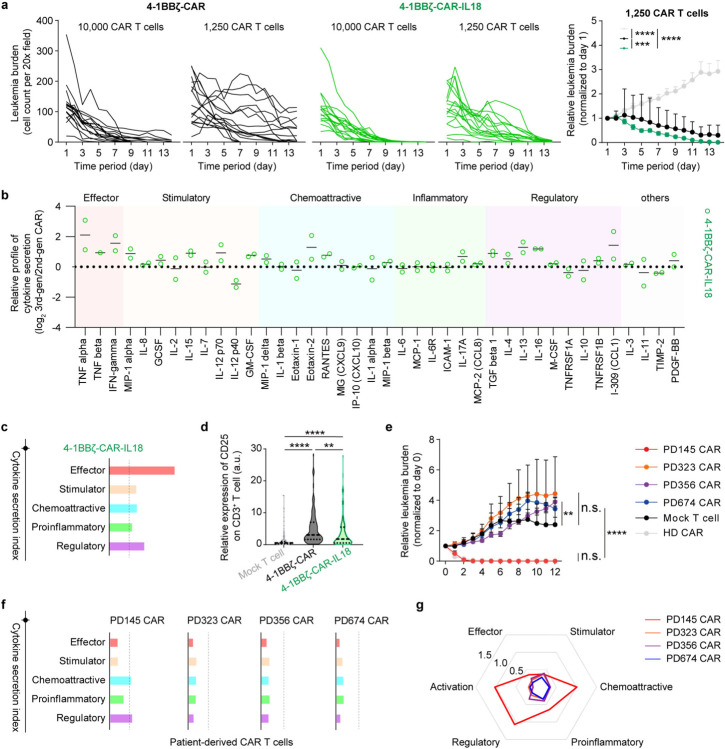
Validating functional performance of CAR T cell products. (**a**) On-chip response curves under treatment of different CAR T cells (4-1BBζ-CAR and 4-1BBζ-CAR-IL18) at the number of 10,000 and 1,250 CAR T cells. Data was collected from four technical replicates (n=4). Data with statistical significance was determined by two-way ANOVA followed by Tukey’s post hoc test, where *P < 0.05, **P < 0.01, ***P < 0.001, ****P < 0.0001, and n.s., not significant (P > 0.05), normalized to 2nd-gen 4-1BBζ-CAR. (**b**) Profiling of cytokine secretion from leukemia chips treated with 3rd-gen 4-1BBζ-CAR-IL18 for 2 days. Data was collected from two independent experiments. (**c**) Cytokine secretion index of 3rd-gen 4-1BBζ-CAR-IL18 benchmarked by 2nd-gen 4-1BBζ-CAR (dash line). Cytokines are divided into five categories, i.e., effector, stimulatory, chemoattractive, inflammatory, and regulatory according to its role in immune response processes and present at weighted average. (**d**) Surface expression of CD25 on different CAR T cells after on-chip interaction with leukemia blasts for 2 days. Data was collected from four technical replicates (n=4). Data with statistical significance was determined by one-way ANOVA followed by Tukey’s post hoc test, where *P < 0.05, **P < 0.01, ***P < 0.001, ****P < 0.0001, and n.s., not significant (P > 0.05). (**e**) Comparative analysis of leukemia burden on-chip treated with different patient derived CAR T cell products. Data was normalized to day 0. Data with statistical significance was determined by two-way ANOVA followed by Tukey’s post hoc test, where *P < 0.05, **P < 0.01, ***P < 0.001, ****P < 0.0001, and n.s., not significant (P > 0.05). (**f**) Cytokine secretion index of different patient derived CAR T cell products, benchmarked by a HD CAR T cell (dash line). (**g**) Radar map depicting functional performance, i.e., activation, effector, stimulator, chemoattractive, proinflammatory, and regulatory, of different PD CAR T cells. PD145 CAR outperformed other PD CAR T cell products.

We then assessed the functionality of 3rd-gen CAR T cell product where along with co-stimulatory signal 4–1BB, another functional domains such as IL-18 is integrated either to potentiate T cell activation or mobilize the bone marrow immunity [52]. On-chip analyses confirmed that 3rd-gen 4-1BBζ-CAR-IL18 achieved a rapid remission response at high dose of 10,000 CAR T cells (Fig. 4a). To reveal functional difference across different CAR designs, we run a ‘CAR stress test’ with low ratios of effector to tumor (2,500 or 1,250 CAR T cells per chip), adapting our recent protocol [53]. The results showed that 2nd-gen 4-1BBζ-CAR took a longer time to eradicate leukemia blasts than did 3rd-gen 4-1BBζ-CAR-IL18 and this difference was further expanded at a lower dose (Fig. 4a and Extended Data Fig. 12). Next, we profiled cytokine secretion from the 3rd-gen CAR T cell and normalized it to that of 2nd-gen CAR T cell. We found that 4-1BBζ-CAR-IL18 demonstrated a robust cytokine secretion upon activation in niche on-chip (Fig. 4b), confirming the role of integration of IL18 to strengthen CAR T cell potency. To better outline the cytokine secretion of CAR T cell, we classified these cytokines into five categories, i.e., effector, stimulatory, chemoattractive, inflammatory, and regulatory, according to its function in immune response processes and quantified the overall performance using weighted average. Again, 4-1BBζ-CAR-IL18 boosted cytokine secretion in all the categories, including those Th2 cytokines (Fig. 4c and Extended Data Fig. 12). This may partially explain why 2nd-gen 4-1BBζ-CAR underperformed 3rd-gen 4-1BBζ-CAR-IL18 during CAR stress test.

Lastly, we evaluated 2nd-gen anti-CD19 CAR T cell products generated from four patients either with B-ALL leukemia (PD323, PD356, and PD674) or non-B-ALL cancer (PD145) using our bioengineered chip (Extended Data Fig. 13a,b). Of these four patients, on-chip remission was only achieved with treatment of PD145 CAR T cells that enhanced surface expression of CD25 at day 2 (Fig. 4d and Extended Data Fig. 13c,d). Also, PD323, PD356 and PD674 CAR T cells demonstrated a significantly weakened cytokine secretion index compared to that of PD145 CAR T cell, benchmarked by a HD CAR T cell (Fig. 4e, Extended Data Fig. 6c, and Extended Data Fig. 13e). The varied outcomes can be partially explained by the limited expansion of T cells from PD323, PD356, and PD674 during CAR T cell manufacturing as on-chip treatments were all given at a dose of 10, 000 CAR T cells (Extended Data Fig. 13b). To better correlate on-chip performance with future clinical outcome, we developed a matrix-based analytical and integrative index to systematically delineate the therapeutic potency of patient CAR T cells where PD145 CAR T cell outperformed other PD CAR T cells (Fig. 4f). Together, our bioengineered leukemia chip demonstrated a useful and powerful platform for CAR T cell development.

## Discussion

The past decade has witnessed unprecedented potential of cancer-on-chip as a novel tool for immune-oncology research. For instance, several studies have developed such platforms to assess cell-based immunotherapies for instance TCR T cell [54,55], CAR T cell [56–58], and NK cell [59–61], as well as their interaction with extrinsic cues such as hypoxia, physical barriers and extracellular matrix, and other immune cells [54–65]. Thus far, the current cancer-on-chip model is in its infancy and an immune-oncology screening platform that can model CAR T cell therapy with unique and complex human immune signatures is highly desirable while largely unavailable. Previously, we reported a leukemia-on-a-chip to study chemoresistance pathways contributed by leukemia bone marrow stroma, and using scRNA-seq we also interrogated healthy and leukemia bone marrow niches and revealed heterogeneous immune dynamics at single-cell level across clinical response scenarios [28–30,37]. Integrating these technical innovations and biological insights, we herein develop a 3D vascularized and immunocompetent leukemia microphysiological system that recapitulates human leukemia bone marrow stroma and immune niches. Replicating the *in vivo* bone marrow microarchitecture and immunity, our leukemia chip allows to map the systematical and functional dynamics of CAR T cell treatment which is at large not offered by conventional *in vitro* assays. Also, we realize to model different therapeutic responses, i.e., remission, resistance, and relapse post CAR T cell therapy observed in clinical settings and map corresponding CAR T cell dynamics, transcending current *in vitro* analyses that are limited to short experimental period and static time points. It is acknowledged that our current leukemia chip setup is capable of reproducing only a few response scenarios that are presented in clinical trials. For example, the on-chip modelled resistance response mainly reproduces clinical refractory cases while the relapse response modelled on-chip is simply testing clonal selection for pre-existing minor CD19^−^ clones that drive relapse upon CAR T cell therapy. Other types of resistance or relapse scenarios such as acquired resistance via surface antigen loss, down-regulation, and mutation following CAR T cell challenge have not been tested on-chip, which can be directions for improvement. Nevertheless, our platform can be assembled rapidly (half day) and allows at least a 14-day long evaluation of CAR T cell responses, which is of great convenience compared to immunocompetent animal models that can take up to half year or longer to establish, let alone month-long experiments subsequently [21–24]. More importantly, based on multi-dimensional and spatiotemporal measurements, we develop a matrix-based analytical and functional index to comprehensively delineate therapeutic functionality of CAR T cell of different designs or generations and confirm that a limited Th2 function may compromise CAR T cell performance as shown in CAR stress test scenario and patient-derived CAR T cell products.

Current attempts to overcome the drawbacks of CAR T cell therapy are primarily focused on persistence and specificity of effector cells. While this is undoubtedly important to maximize the potential of CAR T cell, a detailed understanding of the impact of tumor immunity on CAR T cell therapy is also indispensable [66,67]. To reconstitute the *in vivo* leukemia immune milieu *in vitro*, we populate our leukemia chip with primary human bone marrow mononuclear cells. It is acknowledged that bone marrow mononuclear cells contain less to no granulocytes, thus the regulatory role of these cell types in CAR T cell therapy remains to be investigated in future. Most critically, incorporation of clinical bone marrow samples (primary leukemia blasts and both stroma and immune parts) to build a fully patient-derived screening platform is necessary to enable matched studies between preclinical functional tests and clinical performance of CAR T cell products, though the limited availability of patient samples is definitely a critical issue. Additional work to combine biochemical gradients, hypoxia, ECM stiffness, and interstitial flow into our leukemia chip will further expand our understanding of the effect of other microenvironmental cues on CAR T cell therapy. In conclusion, our tissue engineered immune-oncology model offers an ideal precision medicine platform for preclinical evaluation of CAR T cell immunotherapies, which can accelerate preclinical CAR T cell development, bridge up biological and technical gaps between preclinical studies and clinical trials, and ultimately pave ways to reliably screen responders and non-responders and develop optimal CAR T cell therapy.

## Materials and Methods

### Cell Culture and Reagents

Human B-ALL blasts (Reh, Cat#CRL-8286, ATCC) were cultured in RPMI medium (Cat#11875135, Thermo Fisher Scientific) supplemented with 10% Fetal Bovine Serum (FBS, Cat#A5256701, Thermo Fisher Scientific), 1% penicillin/streptomycin (Cat#15140163, Thermo Fisher Scientific), 100 μM L-glutamine (GlutaMAX Supplement, Cat#35050061, Thermo Fisher Scientific), and 50 μM β-mercaptoethanol (Cat#21985023, Thermo Fisher Scientific) in a 37°C incubator with 5% CO_2_. The Reh B-ALL cell line was validated by short tandem repeat analysis through ATCC. Leukemia cell line was a gift from Dr. Iannis Aifantis’s lab at NYU School of Medicine. GFP stably expressing Reh cell line (Cat#T3959) was bought from Applied Biological Materials and cultured in Prigow IV media (Cat#TM004, Applied Biological Materials) supplemented with 10% FBS and 0.2 μg/mL Puromycin (Cat#G264, Applied Biological Materials). Primary human umbilical endothelial cells (HUVECs, Cat#C2519A, Lonza) and RFP expressing HUVECs (Cat#cAP-0001RFP, Angio-Proteomie) were cultured in Endothelial Cell Growth Medium-2 BulletKit (EGM-2, Cat#CC-3162, Lonza) and used within passage 5. Primary human mesenchymal stem cells (hMSCs, Cat#PT-2501, Lonza) were cultured in Mesenchymal Stem Cell Growth Medium BulletKit (MSCGM, Cat#PT-3001 Lonza) and used within passage 5. Primary normal human lung fibroblast (NHLF, Cat#CC-2512, Lonza) were cultured in Fibroblast Growth Medium-2 BulletKit (FGM-2, Cat#CC-3132, Lonza) and used within passage 8. Osteoblasts were differentiated from hMSCs using Human Mesenchymal Stem Cell Osteogenic Differentiation Medium BulletKit (Cat#PT-3002, Lonza) for at least 12 days and designated as hMSC-derived osteoblasts. Human fetal osteoblastic cell line (hFOB1.19, Cat#CRL-11372, ATCC) was bought from ATCC and validated by short tandem repeat analysis. hFOB1.19 cells were cultured with DMEM/F12 (Cat#11320033, Thermo Fisher Scientific) supplemented with 10% FBS, 1% penicillin/streptomycin, 100 μM L-glutamine, and 0.3 mg/mL G418 (Cat#10131035, Thermo Fisher Scientific). Unless stated otherwise, hFOB1.19 cells were only used for the characterization of the whole scanning of leukemia chip. To create a physiologically relevant bone marrow immune model, commercial bone marrow mononuclear cells (Cat#70001.1, STEMCELL Technologies) containing various bone marrow immune cells from healthy donors were used. Human anti-CD19scFv-4–1BB-CD3ζ CAR (4-1BBζ-CAR, for short) T cells from healthy donors (HD) and leukemia patients (PD) or non-engineered (Mock) T cells are purchased from ProMab Biotechnologies by a customized order with expansion for about 10 days. In addition, 2nd-gen CAR T cells, i.e., CD28ζ-CAR, ICOSζ-CAR, and 4-1BBζ-CAR, and 3rd-gen 4-1BBζ-CAR-IL18, as well as respective Mock T cells, were prepared and shared from Dr. Saba Ghassemi’s lab at University of Pennsylvania School of Medicine. K562-meso-19, K562-meso-19-GFP and K562-meso-19-GFP leukemia cell lines were provided by Dr. Saba Ghassemi’s lab and cultured in RPMI medium supplemented with 10% FBS, 1% penicillin/streptomycin, 100 μM L-glutamine, and 50 μM β-mercaptoethanol. K562-meso-19, K562-meso-19-GFP and K562-meso-19-GFP leukemia cell lines were particularly used for comparatively validating the functional performances of 2nd-gen 4-1BBζ-CAR and 3rd-gen 4-1BBζ-CAR-IL18. Prior to on-chip loading, CAR T cell and Mock T cell were thawed and recovered overnight in ImmunoCult-XF T Cell Expansion Medium (Cat#10981, STEMCELL Technologies) supplemented with 200 U/mL recombinant Human Interleukin-2 (IL-2, Cat#200–02, Peprotech).

### Fabrication of Microfluidic Chip

The 3D microfluidics-based organotypic leukemia bone marrow niche chip is composed of three distinct functional regions (Fig.1 and Extended Data Fig.S1), including a central sinus region vascularized by endothelial cells, an inner ring region that functions as bone marrow cavity populated with bone marrow mononuclear cells, stromal cells and leukemia, and the outer ring channels which serves as endosteal region inoculated with osteoblasts and fibroblasts and connects with four media reservoirs which are for cell culture media supply and waste removal [28–30]. The microfluidic chip was fabricated using a standard soft lithography replica molding technique. The master mold for the microfluidic chip was fabricated with SU-8 negative photoresist (Cat#SU8–2050, Microchem) at a thickness of 100 μm on a silicon wafer (Cat#452, University Wafer) by employing photolithography. Prior to usage, the master mold was surface-modified by trichloro(1H,1H,2H,2H-perfluorooctyl)saline (Cat#448931, Sigma-Aldrich) vapor overnight in a vacuum desiccation to facilitate later polydimethylsiloxane (PDMS) release. Then, a mixture of PDMS base and curing agent (Sylgard184, Cat#DC2065622, Dow Corning) at 10:1 weight-to-weight (*w/w*) ratio were well mixed, cast on the mold, degassed, and then solidified in an 80°C oven for 1 hour. Once PDMS was peeled from the mold, 1 mm, 1.5 mm, and 4 mm holes were punched for two side inlets, one central inlet, and four outlets, respectively. The cleaned PDMS slabs were finally bounded onto glass coverslips (22 × 22 mm, Cat# CLS285022, Thermo Fischer Scientific) to assemble the microfluidic chip using plasma (PE50XL, Plasma Etch; 350 W, 2 minutes) and incubated in an 80°C oven overnight to restore surface hydrophobicity of PDMS chip. Prior to cell loading, microfluidic chips were treated under ultraviolet for sterilization in a Type 2 class laminar flow hood for 20 minutes.

### Preparation of Leukemia Chip

To prepare the leukemia chip model, human leukemia blasts, bone marrow stromal cells, and bone marrow immune cells were loaded into the microfluidic chips with physiologically relevant seeding density for each cell type (i.e., HUVECs at 1×10^7^ cells/mL, hMSCs at 1×10^5^ cells/mL, NHLF at 2×10^6^ cells/mL, hMSC-derived osteoblasts at 2×10^6^ cells/mL, Reh B-ALL at 1×10^6^ cells/mL, and bone marrow mononuclear cells at 5×10^6^ cells/mL) in a fibrin hydrogel. In general, a multiple-step loading protocol were followed to compartmentalize HUVECs in the center of the chip, HUVECs, hMSCs, Reh B-ALL, and bone marrow mononuclear cells in the perivascular area, and NHLF and hMSC-derived osteoblasts in the endosteal region. Unless stated otherwise, CD19 expressing K562 leukemia cell lines were used with a seeding density at 5×10^5^ cells/mL for 3rd-gen CAR T cell testing experiments. First, a sacrificial gelatin (Cat#G6144–100G, Sigma) hydrogel solution of 12 mg/mL in phosphate buffered saline (PBS, Cat#10010049, Thermo Fisher Scientific) was injected into the central area and solidified at −20°C for 15 minutes. This step aims to minimize the generation of bubbles between different regions during the following cell loading process. Then, a mixture of HUVECs, hMSCs, Reh B-ALL cells, and bone marrow mononuclear cells in fibrin solution (3 mg/mL in PBS) containing 2 U/mL thrombin (Cat#604980–100U, Sigma) was infused into the inner ring area and gelled at room temperature for 10 minutes. To recreate the endosteal niche, a mixture of NHLF and hMSC-derived osteoblast in fibrin solution (3 mg/mL in PBS) containing 2 U/mL thrombin was then loaded into the outer ring area by a gentle vacuum suction. Following the gelation, cell culture media was added into the four media reservoirs and the chip was incubated at 37°C for 30 minutes, during which the gelled gelatin will become liquefied and be removed. Finally, HUVECs were seeded to cover the central area and establish interconnection with vessels formed by HUVECs in the inner ring area, which provided vascular openings at the central region. Since multiple types of cells are cultured within a single chip, cell culture media was chosen to mainly support the long-term maintenance of bone marrow immune cells and tissue structure with a mixture of SFEM-II (STEMCELL Technologies), EGM-2, RMPI1640 at a ratio of 1:2:1 and supplement with a cytokine cocktail [Thrombopoietin (TPO, Cat#300–18, Peprotech), Interleukin-6 (IL-6, Cat#200–06, Peprotech), Interleukin-3 (IL-3,Cat#200–03, Peprotech), FMS-like tyrosine kinase 3 ligand (Flt3-L, Cat#300–19, Peprotech) and Stem cell factor (SCF, Cat#300–07, Peprotech), all at 12.5 ng/mL] and recombinant human VEGF-165/VEGFA (Cat#230-00012-10, RayBiotech) at 10 ng/mL. After 7-day culture, the as-prepared leukemia chips were ready for characterization of CAR T cell functionality.

### Scanning Electron Microscopy

Human leukemia bone marrow chips were prepared and cultured for 7 days following the procedure described above. After being washed three times with PBS, the leukemia chips were fixed with 2% glutaraldehyde in PBS (Cat#16010, Electron Microscopy Sciences) for 1 hour. The fixed leukemia chips were then manually cut into slices with thickness of about 2 mm and dehydrated in a graded series of ethanol solutions. In brief, leukemia chip samples were first dehydrated sequentially in 30%, 50%, 70%, 80%, and 90% ethanol for 10 minutes and finally in 100% ethanol three times, each time for 20 minutes. The dehydrated leukemia chip samples were then dried using a CO_2_ Critical Point Dryer (Samdri^®^-PVT-3D, Tousimis) according to the manufacturer’s instruction. The dried leukemia chip samples were mounted on stubs and sputtered with gold palladium for 30 seconds, after which scanning electron microscopy (SEM) images was acquired using a Gemini 300 FESEM with Gatan 3View (Zeiss).

### Time-Lapse Live Microscopy

To clearly visualize and distinguish T cell, leukemia blasts, and niche cells, we labeled CAR T cell or Mock T cell with Vybrant DiD Cell-Labeling Solution (Cat#V22887, Thermo Fischer Scientific) and used Reh leukemia blasts and HUVECs that were genetically engineered to express with GFP (prepared in Dr. Iannis Aifantis’s lab or bought from Applied Biological Materials) or RFP (Angio-Proteomie), respectively. We infused CAR T cells into the leukemia chip via the central sinus and allowed the cells to interact for 2–4 days. After the defined incubation, the chip was mounted on an inverted microscope (Zeiss Axio Observer.Z1) with a motorized stage and an environment control incubation chamber (Okolab) to maintain 37°C with 5% CO_2_. Phase contrast and fluorescent images were recorded every 5 minutes for 12 hours using a digital CMOS camera (ORCA-Flash4.0 LT, Hamamatsu Photonics) with a 20× objective. In addition, single T cell migration was also monitored every 30 seconds in a time period of 4 hours (in total, 481 frames). Cell motility parameters were assessed via tracking of single T cell (>100 cells per conditions) in ImageJ (NIH) using Manual Tracking plug-in where average cell migration speed was defined by the distance traveled in a unit time calculated using the corresponding coordinates at across frames.

### Generation of GFP Expressing and CD19-KO mCherry Expressing Reh B-ALL Cell Lines

GFP expressing and CD19-KO mCherry expressing Reh B-ALL cell lines were generated following the protocol described in our previous study [29]. In brief, Cas9-expressing Reh cell lines were first generated by transduction with retroviral Cas9-2A-blast (Addgene, plasmid no. 73310) and then lentivirally transduced with sgRNAs where control sgROSA (Rosa26: AACGGCTCCACCACGCTCGG) cloned into LRG2.1 (Addgene, plasmid no. 108098) and sgCD19 (CD19_GUIDES_sg459: CCTCATGATTGGGTCCAGGC) into LRCherry2.1 (Addgene, plasmid no. 108099). Following the transfection, mCherry^+^ Reh cells were sorted to generate CD19-KO mCherry expressing Reh cell line and GFP^+^ Reh cells to generated GFP expressing Reh cell line using the SY3200 highly automated parallel sorting (HAPS) cell sorter (Sony). CD19-KO mCherry expressing Reh clone was verified by immunoblots for CD19^−^ before banking for subsequent studies.

### Modeling Remission, Relapse, and Resistance post CAR T cell Therapy on-chip

Clinical diagnosis of leukemia considered leukemia burden in bone marrow below 5% as a remission while 25% as relapse as non-response (relapse and resistance), though PCR or flow cytometry based clinical characterization regarded a lower percentage (0.01–0.1%) of leukemia burden as remission [44–47]. First, we calculated about 10,000 Reh B-ALL cells present in the leukemia after 7-day culture with an initial seeding density at 1×10^6^ cells/mL (approximately 3,000 Reh B-ALL cells, day 0). Treatment with 10,000 CAR T cells (ratio of effector to tumor = 1:1) achieved remission in leukemia chip. Thus, to replicate the resistance scenario, we lowered the ratio of effector to tumor to 1:4, such as 2,500 CAR T cells per chip compared to 10,000 in the remission scenario. To replicate relapse scenario, we spiked CD19^−^ leukemia blasts (1% or 5%) into the whole leukemia population when preparing leukemia chips and estimated 150 of CD19^−^ leukemia blasts per chip (5% condition). Following this setup, we chronologically monitored the leukemia burden (CD19^+^ leukemia with GFP signal while CD19^−^ leukemia with mCherry signal) over 14 days, as well as CAR T cell functionality.

### Collecting and Processing Clinical Data

Clinical data of tumor burdens in leukemia patients during CAR T cell therapy at different time points was adopted and pooled from our recent study [48]. Previously, we collected information of 209 patients from different clinical studies where some studies reported clinical data of individual patients and others provided preprocessed clinical data with only statistical values such as medians. Therefore, we regarded statistical values of different groups (containing one or more individuals) of patients as representative individuals. In brief, publicly available figures from different clinical studies were digitalized with WebPlotDigitizer (https://automeris.io/WebPlotDigitizer) to collect data points which were then converted from peripheral blood to bone marrow if needed and grouped into 14 remission cases, 7 resistance cases, and 11 relapse cases. To plot the curves of leukemia burden of different response scenarios, non-linear fitting was applied to remission and relapse responses and linear regression was applied to resistance response all with 95% confidence intervals using GraphPad Prism.

### Immunostaining

To characterize the cellularity of tissue-engineered bone marrow niche on-chip, leukemia chips were stained with antibody targeting different cell populations, for example, vascular cells (FITC anti-human CD31, Cat#303104, BioLegend), bone marrow mesenchymal stem cells (APC anti-human CD90/Thy1, Cat#328113, BioLegend), hematopoietic cells (APC anti-human CD45, Cat#304012, BioLegend), T cells (CD3, FITC anti-human CD3, Cat#317306, BioLegend, PE anti-human CD3, Cat#317308, BioLegend, and APC anti-human CD3, Cat#300412, BioLegend; CD4, PE anti-human CD4, Cat#317410, BioLegend; CD8, FITC anti-human CD8a Cat#300906, BioLegend, PE anti-human CD8a Cat#300908, BioLegend, APC anti-human CD8a, Cat#300912, BioLegend), monocytes (Alexa Fluor 647 anti-human CD14, Cat#325611, BioLegend), and macrophages (Alexa Fluor 488 anti-human CD68, Cat#333812, BioLegend), and hematopoietic stem cells (APC anti-human CD34, Cat#343510, BioLegend).

To quantify the deposition of extracellular matrices during the 7-day culture, leukemia chips were fixed with Fixation Buffer (Cat#420801, BioLegend) each day, and stained with DyLight488-Laminin (Cat#PA522901, Thermo Fisher Scientific), PE-Fibronectin (Cat# IC1918P, R&D Systems), and Alexa Fluor 647-Collagen IV (Cat#51-9871-80, Thermo Fisher Scientific) all at dilution of 1:50.

To quantify T cell activation on-chip, leukemia chips were first incubated with FITC anti-human CD3 (1:20, Cat# 317306, BioLegend), PE anti-human CD69 (1:50, Cat#310906, BioLegend), or PE anti-human CD25 (1:50, Cat#302606, BioLegend), as well as Human TruStain FcX (1:50, Cat#422301, BioLegend) for block of Fc Receptor for about 4 hours at 4°C, then thoroughly washed for three times with PBS followed by fixation using FluoroFix Buffer (Cat#422101, BioLegend) for 20 minutes. To stain intracellular granzyme B (PE anti-human granzyme B, Cat#372208, BioLegend) or Ki67 (APC anti-human Ki67, Cat#350514 or Alexa Fluor 488 anti-human Ki67, Cat#350508, BioLegend), leukemia chips were first permeabilized with 0.3% Triton X-100 (Cat#11332481001, Sigma-Aldrich) for 20 minutes, and then thoroughly washed for three times after incubation with antibodies at dilution of 1:50 for 4 hours at room temperature or overnight at 4°C. To indicate CAR T cell, leukemia chips were first incubated with biotinylated monoclonal anti-FMC63 scFv (CAR) antibody (1:50, Cat#FM3-BY54, ACRO Biosystems), followed by incubation with PE (Cat#405245, BioLegend) or APC (Cat#405243, BioLegend) conjugated Streptavidin (1:50). The leukemia chips were finally incubated for 10 minutes with 4’,6-diamidino-2phenylindole (DAPI, Cat#D1306, Thermo Fisher Scientific) to counterstain nuclei.

To quantify the activation of bone marrow niche cells, Alexa Fluor 647 anti-human CD14 (Cat#325612, BioLegend) and PE anti-human HLA-DR (Cat#307606, BioLegend) were used to stain HLA-DR expression on CD14^+^ monocytic cells, whereas Alexa Fluor 488 anti-human CD54 (ICAM-1, Cat#353129, BioLegend) and PE anti-human CD31 (Cat#303106, BioLegend) or PE anti-human VE-cadherin (Cat#348506, BioLegend) were used to characterize expression of ICAM-1 on vascular cells.

All the stained samples were kept in Cell Staining Buffer (Cat#420201, BioLegend) and imaged with Nikon C2i confocal microscope unless stated otherwise. The obtained images were processed in Nikon NIS-Elements Microscope Imaging Software and mean intensity of each maker was quantified using ImageJ (NIH) or Fiji.

### Flow Cytometry

T cell activation marker was detected using FITC anti-human CD3 (1:20), PE anti-human CD69 (1:50), PE anti-human CD25 (1:50), PE anti-human GZMB (1:50), and Human TruStain FcX (1:50). Single-cell suspensions of leukemia chip samples (CAR and Mock) were first prepared by off-chip recovery with nattokinase (50 Fu/mL, NSK-SD, Japan Bio Science Laboratories) [28–30], and then respectively incubated with antibodies for 30 minutes at 4°C. Cells were washed three times and stained with LIVE/DEAD Fixable Aqua Dead Cell Stain Kit (1:1500, Cat#L34957, Thermo Fischer Scientific) to mark live/dead cells. All samples were analyzed via a LSRII UV Flow Cytometer (BD Biosciences) and processed via FlowJo version 10 (Treestar, BD Biosciences). Fluorescence compensations were prepared by incubating respective antibodies with CompBead Anti-Mouse Ig, κ/Negative Control Particles Set (Cat#552843, BD Biosciences). All T cells were gated as live CD3^+^ population (Extended Data Fig. 14).

### Profiling and Quantification of Cytokine Secretion

Qualitative profiles of cytokine secretion from leukemia chips were examined by using a Human Inflammation Array C3 membrane kit (Cat#AAH-INF-3–8, RayBiotech) according to the manufacturer’s protocols. In brief, supernatants were collected from 2–4 leukemia chips after on-chip infusion of CAR T cell and Mock T cell for certain time points, centrifuged at 13,000 r/min for 20 minutes at 4°C to remove cellular debris, and then incubated overnight with Human Inflammation Array membranes (or stored at −20°C for future assays). Biotinylated Antibody Cocktail was incubated with the membranes at 4°C overnight, followed by washing three times and incubation with HRP-labeled Streptavidin at 4°C overnight. The mixture of Detection Buffer C and D was then applied for 2 minutes to visualize chemiluminescence at room temperature. Imaging was obtained by using a ChemiDoc Imaging System (Bio-rad). Mean intensity of each spot was quantified in ImageJ (NIH) or Fiji using Protein Array Analyzer plug-in (written by Gilles Carpentier, Faculté des Sciences et Technologies, Université Paris, Paris, France).

Absolute concentrations of different cytokines were measured with respective enzyme-linked immunosorbent assay (ELISA) kits, such as Human IFN-γ ELISA MAX Deluxe (Cat#430104, BioLegend), Human IL-10 ELISA MAX Deluxe (Cat#430604, BioLegend), Human IL-13 Uncoated ELISA Kit (Cat#88-7439-22, Thermo Fisher Scientific), Human Perforin ELISA Set (Cat#ab83709, Abcam), Human granzyme B Set (Cat#DY2906–05, R&D Systems), and Human CCL1/I-309 DuoSet (Cat#DY272, R&D Systems), according to the manufacturer’s protocols.

### scRNA-seq Analysis

The leukemia samples (designated as HD CAR, PD CAR, Mock, None) were engineered and cultured on-chip for 7 days as described above followed by 2-day treatment with CAR T cell, PD CAR T cell, Mock T cell or left untreated. Single-cell suspensions of leukemia samples were prepared by off-chip recovery with nattokinase, pre-labeled with different anti-human hashtag antibodies (TotalSeq, BioLegend), i.e., Hashtag1-GTCAACTCTTTAGCG, Cat#394601; Hashtag2-TGATGGCCTATTGGG, Cat#394603, Hashtag3-TTCCGCCTCTCTTTG, Cat#394605, Hashtag4-AGTAAGTTCAGCGTA, Cat#394607, and mixed into one sample. Correspondingly, freshly thawed HD CAR T cell, PD CAR T cell, Mock T cell, and bone marrow mononuclear cells were pre-labeled with different anti-human hashtag antibodies, mixed into one sample, and used controls. The libraries were prepared using the Chromium Single Cell 3′ Reagent Kits (v3): Single Cell 3′ Library & Gel Bead Kit v3 (PN-1000075), Single Cell 3′ Chip Kit v3 (PN-1000073) and i7 Multiplex Kit (PN-120262) (10x Genomics) and following the Single Cell 3′ Reagent Kits (v3) User Guide (manual part no. CG000183 Rev B). Libraries were then run on an Illumina NovaSeq 6000 using 28bp read 1, 8bp i7 index, and 91bp read 2.

The raw sequencing results from the 10x Genomics platform were processed using CellRanger (version 3.1). In brief, CellRanger was used to generate a count matrix by aligning output reads, filtering the empty dropouts and counting unique molecular identifiers (UMIs). We used Ensemble hg38/GRCh38 as the reference genome for read alignment. Further analyses including quality control and data filtering, the identification of highly variable genes, dimensionality reduction, standard unsupervised clustering algorithms, and the discovery of differentially expressed genes were performed using the R package Seurat (version 4.1.1) [68].

To visualize the data, the dimensionality of the scaled integrated data matrix was reduced to project the cells in two-dimensional space using principal component analysis (PCA) followed by uniform manifold approximation and projection (UMAP) (https://umap-learn.readthedocs.io/) based on 40 PCs with 30 nearest neighbors used to define the local neighborhood size with a minimum distance of 0.3 for the datasets [69]. The resulting PCs were also used as a basis for partitioning the dataset into clusters using a smart local moving (SLM) community detection algorithm (https://www.ludowaltman.nl/slm/) with 30 nearest neighbors for the datasets. A range of resolutions (0.1–10) was utilized to establish a sufficient number of clusters to separate known populations based on the expression of established markers. Cell clusters were annotated based on differentially expressed genes (DEGs) and published marker genes for cell types. To find markers that define individual clusters, we performed pairwise differential expression analysis using the Wilcoxon rank sum test with Bonferroni multiple-comparison correction for each cluster against all other clusters for genes that were detected in at least 10% of the cluster cells, keeping the genes that were significant in each of the comparisons (fold-change difference >10% with adjusted p-value < 0.01).

### Statistics

All the results, including error bars in the graphs, are shown as mean ± standard deviation (SD). The biological and technical replicates and repetitions for each experiment are listed in the respective figure legends. A significant difference between the two groups was determined by unpaired two-tailed Student’s t-test with GraphPad Prism software (version 10), as indicated in the figure legends. Multiple groups were compared by one-way or two-way analysis of variance (ANOVA) as indicated in the figure legends. Statistical significance is defined as follows: *P < 0.05, **P < 0.01, ***P < 0.001, ****P < 0.0001, and n.s. (not significant; P > 0.05).

## Extended Data

**Extended Data Fig. 11.**
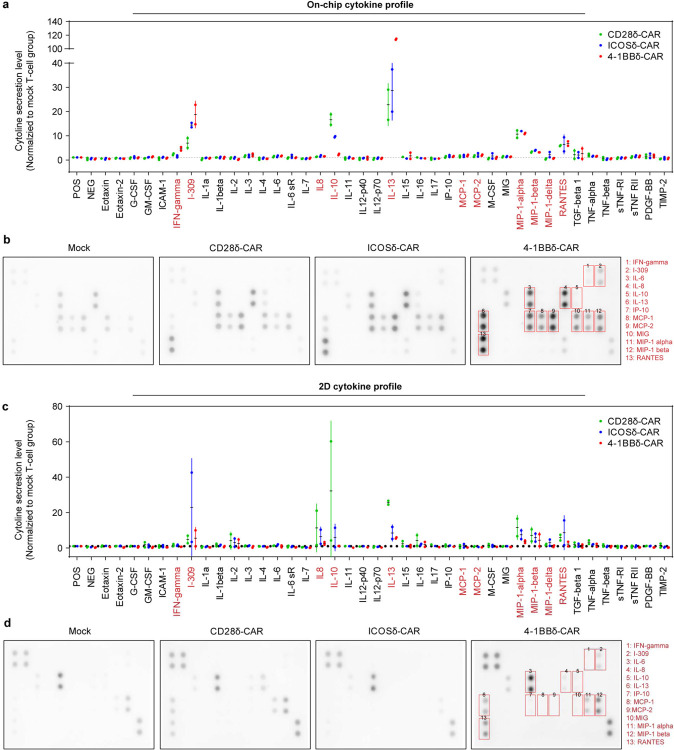
Cytokine secretion profiling of 2nd-gen CAR T cell products. Cytokine secretion profiles of Mock T cell, 2nd-gen CD28ζ-CAR, ICOSζ-CAR, and 4-1BBζ-CAR from either (**a**,**b**) on-chip or (**c**,**d**) 2D co-culture conditions at day 2 were examined by using a Human Inflammation Array C3 membrane kit. Data was from two healthy donors (ND164 and ND365).

**Extended Data Fig. 12.**
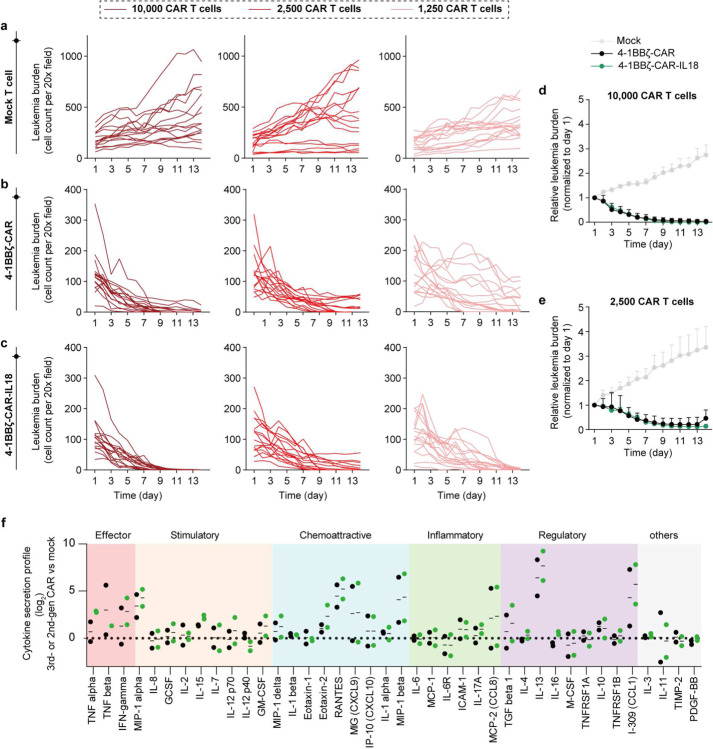
On-chip response curve under treatment of different generation CAR T cells at different doses. (**a**) Mock T cell. (**b**) 2nd-gen 4-1BBζ-CAR. (**c**) 3rd-gen 4-1BBζ-CAR-IL18. Data was collected from four technical replicates (n=4). (**d**,**e**) Comparative results of on-chip response curves under treatment of different CAR T cells at the number of (**d**) 10,000 and (**e**) 2,500. (**f**) Cytokine secretion profiles of Mock T cell, 2nd-gen 4-1BBζ-CAR, and 3rd-gen 4-1BBζ-CAR-IL18 on-chip at day 2 were examined by using a Human Inflammation Array C3 membrane kit. Data from two healthy donors, ND410 (left column) and NM 11/03 (right column). Quantified data, corresponding to Fig. 4b,c.

**Extended Data Fig. 13.**
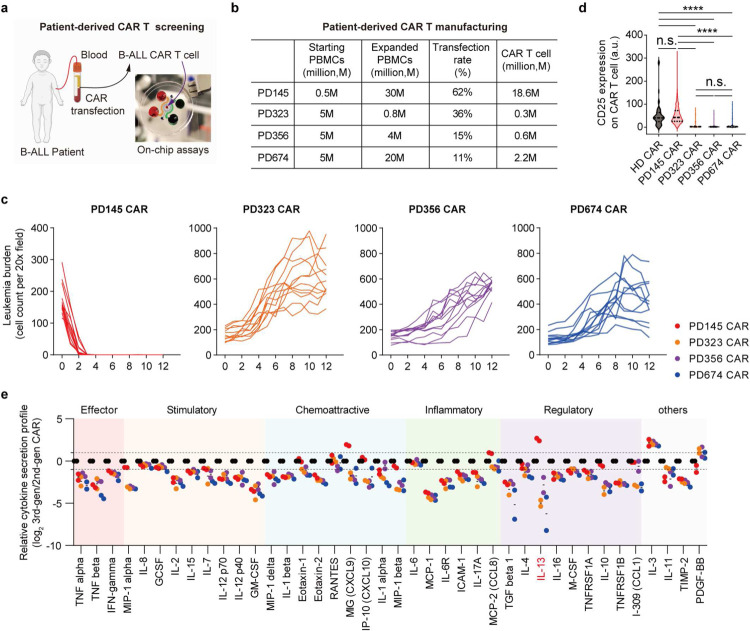
On-chip response curves under treatment of different patient derived CAR T cells. (**a**) Schematic of on-chip evaluation of patient derived CAR T cell products. (**b**) Manufacturing of patient derived CAR T cells, where three patients with B-ALL leukemia (PD323, PD356, and PD674) and one control patient with lung cancer (PD145). (**c**) On-chip response curves under treatment of different patient derived CAR T cells, PD145 CAR, PD323 CAR, PD356 CAR, and PD674 CAR. Data was collected from at least technical replicates (n≥3). (**d**) Surface expression of CD25 on patient derived CAR T cells, PD145 CAR, PD323 CAR, PD356 CAR, and PD674 CAR, after on-chip interaction for 2 days. Data was collected from at least technical replicates (n≥3). Data with statistical significance are as indicated: *P < 0.05, **P < 0.01, ***P < 0.001, ****P < 0.0001, and n.s., not significant (P > 0.05). (**e**) Cytokine secretion profiles of patient-derived CAR T cell products, PD145 CAR, PD323 CAR, PD356 CAR, and PD674 CAR, on-chip at day 2 were examined by using a Human Inflammation Array C3 membrane kit. Data from two independent experiments, corresponding to Fig. 4f.

**Extended Data Fig. 14.**
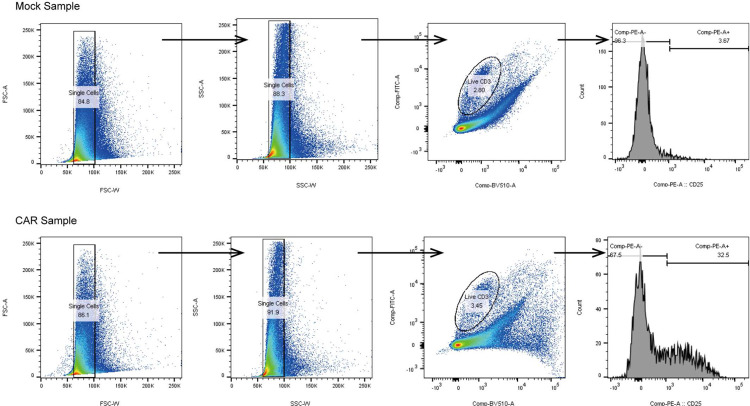
Gating strategy for identification of live CD3^+^ T cell.

## Data Availability

Data supporting the results in this study are available within the paper and its Supplementary Information. The raw and analyzed datasets generated during the study are available from the corresponding author on reasonable request. Source data are provided with this paper. The scRNA-seq data is available in the Gene Expression Omnibus (GEO) under accession number GSE138811.
